# Maxillary segmental osteoperiosteal flap with simultaneous placement of dental implants: case report of a novel technique

**DOI:** 10.1186/s40729-017-0067-5

**Published:** 2017-01-19

**Authors:** Tibebu Tsegga, Thomas Wright

**Affiliations:** 1 Department of Oral & Maxillofacial Surgery, San Antonio Military Medical Center, 3551 Roger Brooke Dr., Ft. Sam Houston, 78234 TX USA; 2 Department of Oral & Maxilofacial Surgery, Wilford Hall Ambulatory Surgical Center, 2200 Bergquist Dr, Suite 1, Lackland AFB, TX 78236 USA

## Abstract

Dental restorative space from the opposing dentition requires adequate distance for restorative material for an acceptable restoration. Typically, long-standing edentulous alveolar ridges will have vertical and or horizontal defects that require alveolar ridge augmentation for ideal dental implant restorations. Along with these defects, one will see the opposing dentition supra erupt which can obliterate the restorative space. Multiple surgical techniques have been described to address these dilemmas. The use of osteoperiosteal flaps has been described to address vertical height deficiencies. The purpose of this paper is to document and introduce a maxillary segmental osteoperiosteal flap intrusion to increase the restorative space with simultaneous dental implant placement. As with most dilemmas in treatment planning dental implants, multiple acceptable treatment options are available to the practitioner. This technique is another of many that can be added to the available options. When appropriately planned in select cases, this technique will result with ideal dental implant restorations without compromising the esthetic and functional harmony of the native dentition.

## Background

Obtaining proper occlusal clearance to allow for a single unit crown restoration is a fundamental prerequisite for dental implant restoration. Long-standing edentulous sites are often fraught with disuse atrophy and unopposed supra-eruption of the opposing dentition. In the posterior maxillae/mandible, there are vital structures that have to be mobilized in order to allow space for either bone transposition or onlay/inlay grafting. There are several predictable techniques described to address these preprosthetic alveolar deficiency dilemmas [1–3].

The osteoperiosteal flap technique has made a strong contribution towards management of these defects. Mobilizing a segment of alveolus attached to the overlying soft tissue can obtain uni- or bi-directional augmentation. This case report describes an amplification of a vertical osteoperiosteal flap with concomitant placement of dental implants in a partially edentulous dental arch.

## Case Presentation

**Fig. 1:**
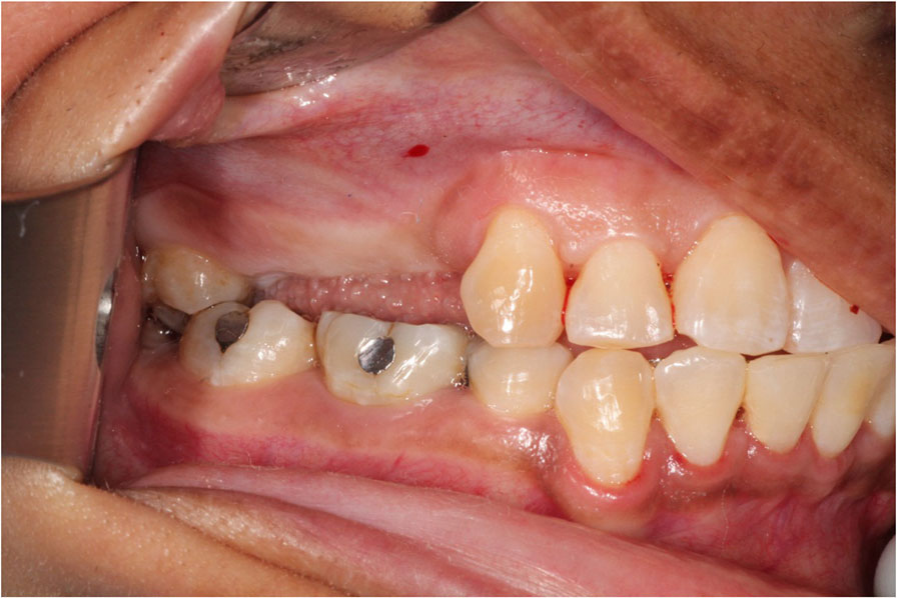
Edentulous site with supra-eruption of opposing dentition

**Fig. 2:**
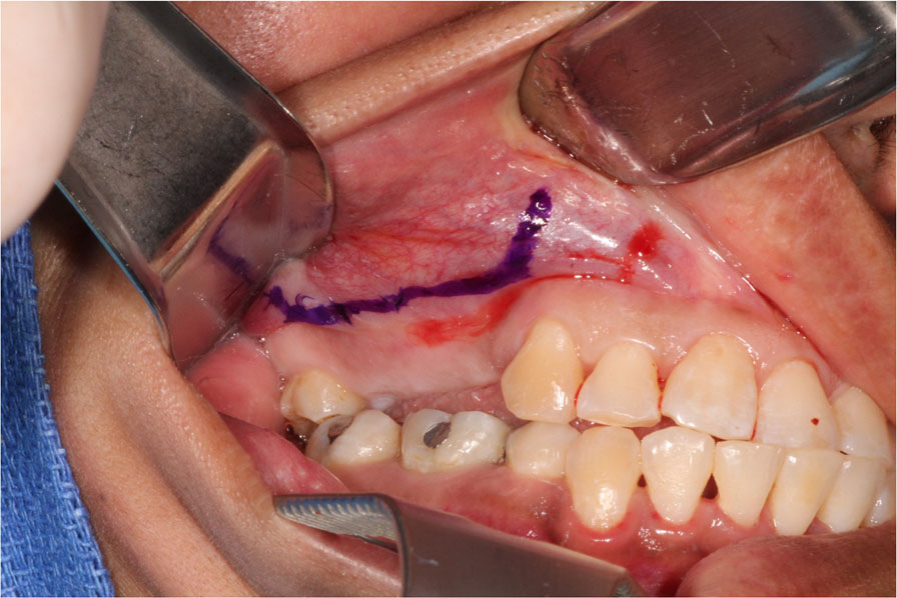
Marked incision site for surgical access

**Fig. 3:**
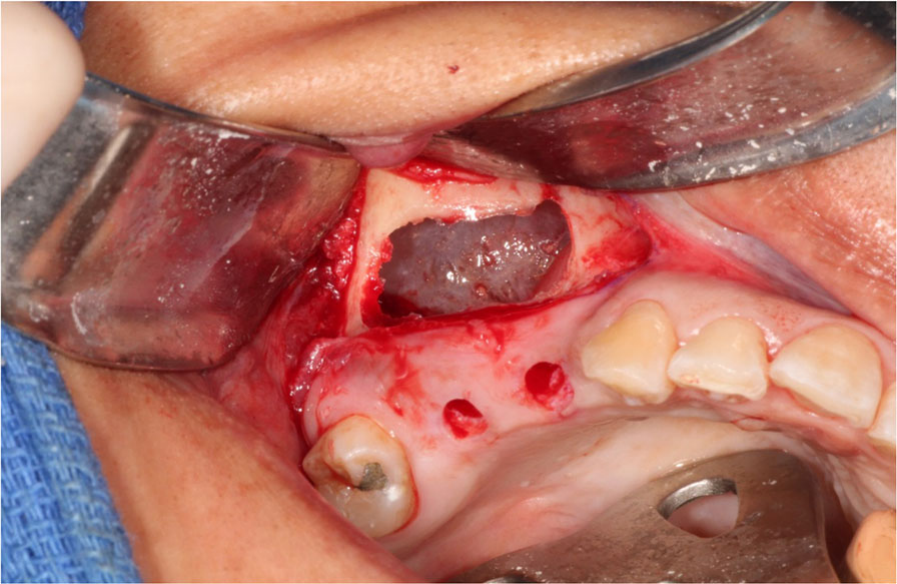
Direct sinus lift with implant osteotomy preparation

**Fig. 4:**
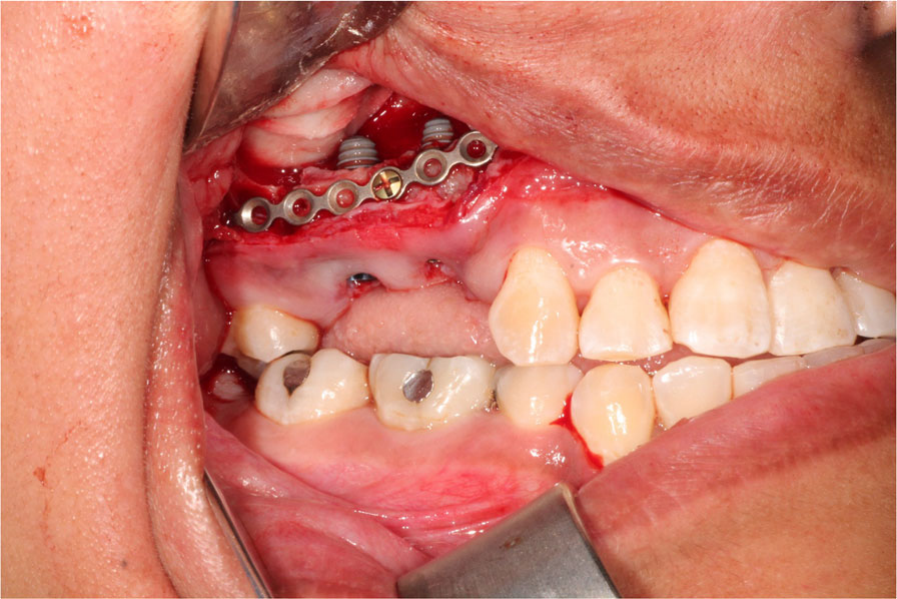
Vertical repositioning of dental alveolus segment with placement of dental implants

**Fig. 5:**
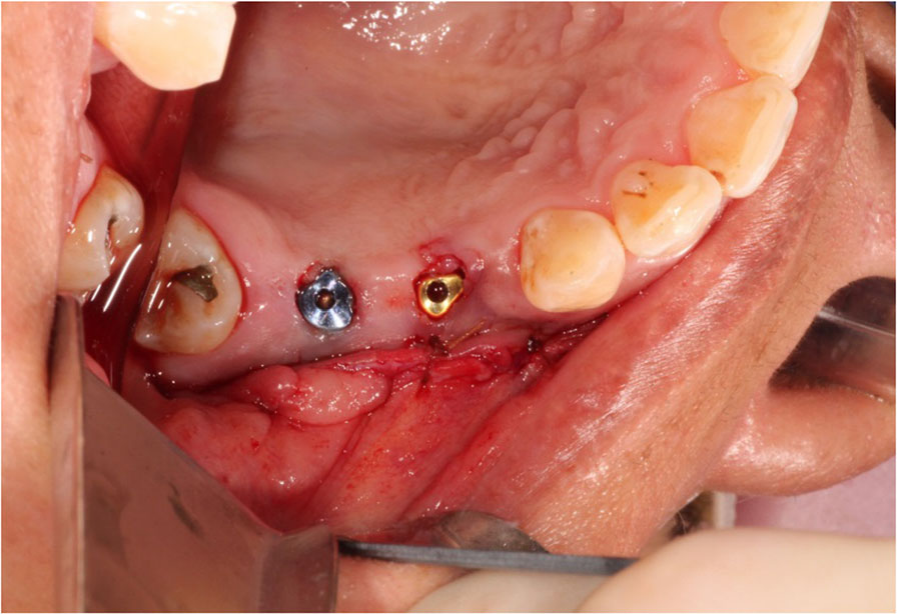
Occlusal view of implants after vertical repositioning of the dental alveolus segment showing proper mesiodistal space and buccolingual spacing

**Fig. 6:**
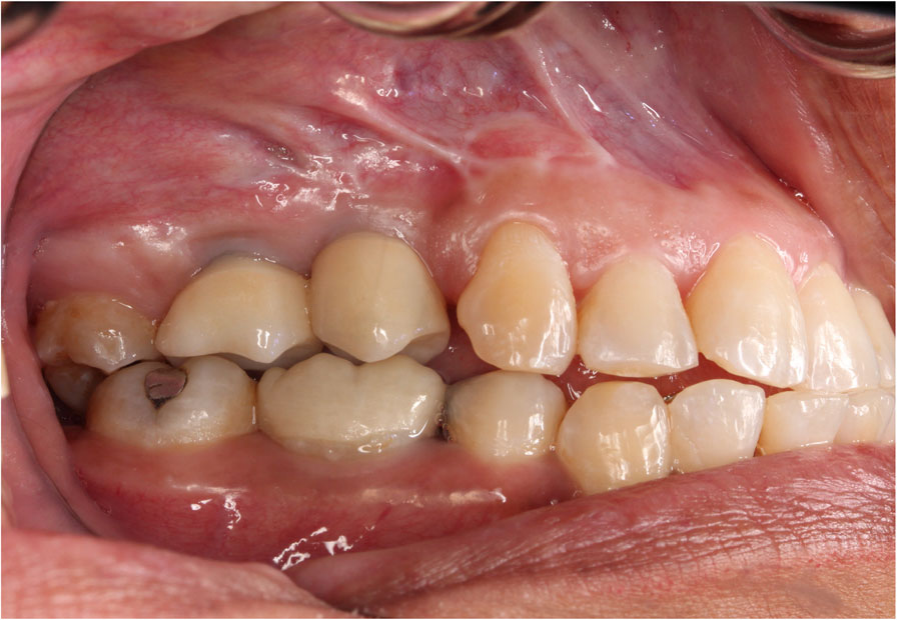
Clinical picture 2 years after implant placement

**Fig. 7:**
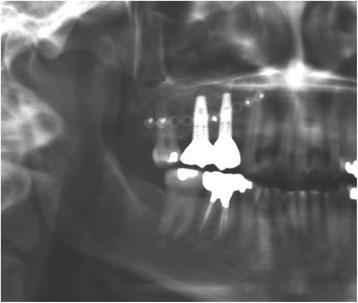
Orthopantomograph 2 years after implant placement

A 35-year-old female with a 10-year history of partial acquired edentulism at site numbers 3 and 4 presented to our clinic for dental implant evaluation. Preoperative clinical examination revealed a reproducible intercuspation, well-delineated band of keratinized tissue, and decreased inter-occlusal clearance to allow for optimal dimension of prosthetic crowns (Fig. 1). Radiographs demonstrated excessive pneumatization of the antrum in the respective area. The preoperative planning included fabrication of two surgical splints. The first splint was fabricated for transmucosal positioning of the implant osteotomy sites in the existing alveolus position. The second splint was fabricated from the predetermined augmented vertical position of the dentoalveolar segment with ideal inter-occlusal clearance. Our surgical treatment began with a horizontal incision 3 mm apical to the mucogingival junction, a full thickness mucoperiosteal flap was created exposing the anterior and posterior boundaries of the proposed segmental osteotomy (Fig. 2). Similar to alveolar distraction techniques, minimal mucosa was elevated off of the transport or movable segment to maintain adequate blood supply. A lateral sinus window technique was used to access the antrum, and the associated Schneiderian membrane was elevated and completely cleared from all boundaries of the respective dentoalveolar segment (Fig. 3). A right angle piezosurgery blade (Piezosurgery Inc., Piezosurgery3 Unit, OT1 insert, OT2 insert) was used to initially create the horizontal/apical osteotomy, which was followed by crestally diverging full thickness vertical osteotomies at the mesial/anterior and distal/posterior areas of the edentulous dentoalveolar segment at site numbers 3 and 4. Before mobilization of the osteoperiosteal flap, the predetermined implant osteotomies were made using the initial surgical splint, and the respective implants (Nobel Biocare, NobelReplace Tapered Groovy) were placed into the predetermined location. Mobilization of the osteoperiosteal flap with a T-handle osteotome confirmed successful separation from the maxillae proper. With the sinus membrane lifted and protected, the vertical repositioning of the osteoperiosteal flap with the positioned implants was accomplished using the second prefabricated splint. In an effort to control torque movement of the mobile segment, we placed the implant placement driver and with the shaft coming through the pilot drill holes of the second guide. The mobile segment was then secured to the anteriorly and posteriorly intact lateral wall of the antrum using an eight-hole 0.6 mm profile curvilinear plate (KLS Martin 1.5 mm, 0.6 mm profile) (Fig. 4). The region under the lifted sinus membrane was then packed with mineralized allograft (Medtronic Sofamor Danek, 0.6–1.25 mm cortical and cancellous chips) in a routine manner. A resorbable membrane (Geistlich Bio-Gide) was then placed over the grafted sinus and fixation mini-plate. The platform of the respective transmucosal placed implants were tactically interrogated to confirm approximation with the alveolar crest. The cover screws were then placed (Fig. 5), and the patient underwent a 4-month healing period. Normal progression to healing abutments and final prosthesis was accomplished (Fig. 6). Pt was followed up 2 years after loading of the implant without any untoward sequelae and radiographic evidence of osseointegrated dental implants (Fig. 7).

## Discussion

A suitable alternative surgical management of this particular case might have been to simply perform an alveoloplasty to produce the desired inter-occlusal clearance and proceed with placement of implant and simultaneous direct sinus lift. That would have left more of the apical portion of the implant within the grafted sinus and possibly modified the location of keratinized band of tissue. The location of the dental alveolar segment in relation to any antral septae also needs to be appreciated, as this described technique can be fraught with complication if such anatomical obstacles are not accounted for preoperatively [4].

The osteoperiosteal flap or “bone flap” commonly used in segmental orthognathic surgery is a bone fragment moved in space without detachment of the investing periosteum [5]. The prerequisite for simultaneous implant placement in a vertical repositioning bone flap is adequate width within the transport segment. It is always a fine balance between allowing enough exposure to place the fixation device without significantly compromising periosteal vascular input into the bone segment. As it is well documented both clinically and experientially, full thickness mucoperiosteal releases will cause some degree of bone resorption at the labial plate [6].

Due to the presence of fixation plate and a sizeable sinus window, we decided to use a long-lasting resorbable membrane. In our experience and supported by the literature, placement of a membrane over the osteotomy site has been shown to increase the amount of bone formation [7]. Considering we were only able to obtain one monocortical screw fixation on the mobilized portion of the maxillae, maintaining immobility during the critical phase of bone healing was an obvious liability. Animal studies which have investigated the biology of small segment wound healing have noted that after 2 weeks, revascularization of the small dento-osseous segment was noted [8]. The cross application of such animal studies are helpful but do not completely capture the additional challenges in this case report. The studies in animals were looking at segmental dental alveolar segments which encompassed the natural teeth. In our case illustration, there were osteotomies made within the transport segment and healing of the overlying particulate allograft was contingent on biological stability of the respective segment. This is a clear illustration of how animal models can begin to provide a platform towards technical innovation, but there is always a parameter of uncharted terrain in translating to human clinical application.

A critical appraisal of the gingival architecture in the final end point of this case demonstrates some radiolucency through the soft tissue outlining the platform of the Nobel Biocare TiUnite implant. This would lead us to believe that either the transmucosal bone level placement attempt was inaccurate or excessive reflection of the labial tissue has caused some degree of resorption. This is another liability that needs to be carefully addressed if this application is recaptured within the esthetic zone. Perhaps slight subcrestal placement of the dental implant or platform switched body feature would minimize this outcome. In our application, we utilized an implant platform topography that is purported by the manufacture to allow soft tissue adhesion and minimize crestal bone loss.

## Conclusions

This case highlights the evolving variations in dentoalveolar augmentation with an emphasis on concomitant implant placement. In the most traditional sense, a vertical osteoperiosteal flap technique would be bound with a stable basal bone that can be used to anchor simultaneous dental implant placement. Further refinement should consider minimizing crestal reflection and overall labial bone resorption.
